# Pembrolizumab-induced acute thrombosis: A case report

**DOI:** 10.1097/MD.0000000000010772

**Published:** 2018-05-18

**Authors:** Kei Kunimasa, Kazumi Nishino, Madoka Kimura, Takako Inoue, Motohiro Tamiya, Toru Kumagai, Fumio Imamura

**Affiliations:** Department of Thoracic Oncology, Osaka International Cancer Institute, Otemae Chuoku, Osaka City, Japan.

**Keywords:** acute thrombosis, pembrolizumab, pulmonary artery thromboembolism

## Abstract

**Rationale:**

Acute thrombosis has not been reported in the literature so far in lung cancer patients as an immune-related adverse event (irAE) associated with PD-1 pathway inhibitors.

**Patients concerns:**

Here, we for the first time present two NSCLC (non-small cell lung cancer) patients suffering from acute thrombosis as a pembrolizumab-induced irAE. Immediate treatment with continuous heparin infusion improved their symptoms and enabled them to continue pembrolizumab administration.

**Methods:**

Ethical approval was given by the ethics committee of Osaka International Cancer Institute and the informed consents were given by the patients.

**Diagnosis:**

Serum D-dimer level testing, venous ultrasonography, enhanced computed tomography (CT).

**Interventions:**

Continuous heparin infusion, direct oral anticoagulants (DOAC).

**Outcomes:**

Immediate continuous heparin infusion improved their symptoms and continuing pembrolizumab with direct oral anticoagulant successfully induced tumor shrinkage.

**Lessons:**

Reinvigoration of exhausted T cells by pembrolizumab induced systemic inflammation possibly resulting in development of thrombosis. Although acute thrombosis is a rare irAE, it may lead to cessation of treatment and can be lethal.

## 1 Introduction

Pembrolizumab is a humanized monoclonal immunoglobulin (Ig) G4 antibody directed against human cell surface receptor PD-1 (programmed death-1 or programmed cell death-1). It showed a significantly longer progression-free and overall survival than conventional platinum doublets in advanced NSCLC (non-small cell lung cancer) with high PD-L1 protein expression in the first-line setting.^[1]^ Although overall safety profile of pembrolizumab appeared to be better, infrequent but severe immune-mediated adverse events (irAEs) occurred in pembrolizumab group.

So far, thrombosis have not been reported as an side effect of pembrolizumab in lung cancer.^[2]^ Here, we report a case of acute thrombosis associated with pembrolizumab in non-small cell lung cancer (NSCLC) patient. Pulmonary embolism resulted from deep vein thrombosis (DVT) is often life-threatening,^[3]^ and therefore we should pay attention to thrombotic adverse events of immune check point inhibitors.

## 2 Methods

Ethical approval was given by the ethics committee of Osaka International Cancer Institute and the informed consents were given by the patient.

## 3 Results

### 3.1 Case presentation

**Figure 1:**
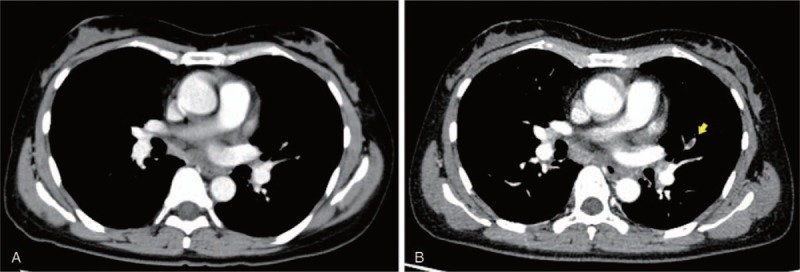
Chest-enhanced CT images; (A) Before pembrolizumab administration (B) On day 7 after administration. Yellow arrow indicates enhancement defect suggesting thrombus formation in the left pulmonary artery. CT = computed tomography.

**Figure 2:**
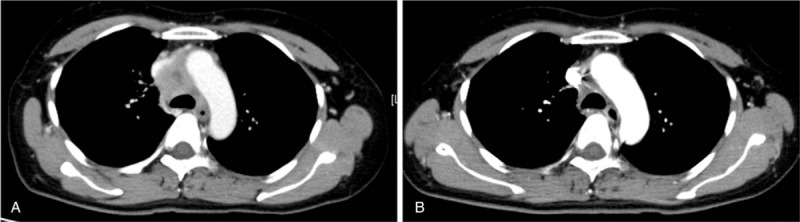
Chest-enhanced CT images; (A) Before pembrolizumab administration (B) After 3 courses of administration. CT = computed tomography.

A 48-year-old, never-smoking woman presented to our hospital complaining of left cervical lymphadenopathy and left shoulder pain. She had been healthy and had no known allergic history and family history. A computed tomography (CT) scan revealed a lung mass invading to the aorta and multiple mediastinal lymph node swelling. Surgical biopsy of a left cervical lymph node revealed adenocarcinoma. After a staging workup with enhanced brain MRI and PET-CT, she was diagnosed as lung cancer of clinical stage IV (cT3N3M1c) with multiple brain metastases. *EGFR* mutation, *ALK* and *ROS1* fusion genes were not detected. PD-L1 immunohistochemistry using PD-L1 22C3 pharmDx revealed the tumor PD-L1 proportion score (TPS) ≥ 90%. Coagulation tests are within normal limit including complete blood count, Factor V assay, fibrinogen level and prothrombin time. As the first-line chemotherapy, pembrolizumab was administered at a dose of 200 mg every 3 weeks. On day 7 of the first course, she felt pain and numbness in her left lower leg and visited our hospital urgently. Venous ultrasonography of her lower limbs demonstrated deep vein thrombosis, which had not been detected before pembrolizumab administration. Furthermore, enhanced chest CT revealed a thrombus in pulmonary artery, leading to the diagnosis of acute thromboembolism (Fig. 1). Serum D-dimer level increased from 6.9 to 33.5 μg/mL. Continuous infusion of heparin was initiated for resulting in improvement of her symptoms in 7 days. Heparin infusion therapy was changed to apixaban; one of direct oral anticoagulants (DOACs). Pembrolizumab, which had been temporarily stopped, was re-started with apixaban. Continuing pembrolizumab with apixaban showed a favorable clinical effect (Fig. 2) and no recurrence of thrombosis was observed.

## 4 Discussion

The antitumor effect of PD-1 pathway inhibitors is mainly due to reinvigoration of exhausted PD-1(+) T cells,^[4]^ which also induces irAEs in more than 20% of the patients treated with them. These irAEs are usually mild and easily manageable in most cases.^[5]^ In this report, we presented a NSCLC patient suffered from acute thrombosis induced by pembrolizumab. Although acute thrombosis is rare and unreported in association with pembrolizumab, it may lead to cessation of treatment and can be lethal.

A combination of blood stasis, plasma hypercoagulability, and endothelial dysfunction is thought to trigger thrombosis.^[3]^ There has been a growing understanding of the central role of inflammation on the local fibrinolytic-thrombotic balance in the initiation of local vascular thrombosis.^[6,7]^ PD-1 pathway inhibitors unleash exhausted T cells in tumors and the reinvigorated T cells evoke inflammation. Reinvigorated PD-1(+) T-cell response to anti-PD-1 therapy in peripheral blood peaks at 3rd week after the initiation of treatment.^[4]^ Thrombosis as an irAE can be associated with the surge of reinvigorated T cells soon after pembrolizumab administration. The present case developed acute thrombosis in the relatively early phase, on day 7 of the first course. This could reflect early phase inflammation induced by pembrolizumab.

Coagulation disorders including thrombosis are common in cancer patients as represented by Trousseau’ syndrome.^[8]^ Although the primary approach to treating hypercoagulopathy associated with cancer is eliminating the causative tumor, heparin is a preferred alternative, which has multiple moderating actions in the coagulation cascade.^[8]^ Specific blocking of factor Xa or thrombin has little data on the efficacy and safety for the treatment of cancer-associated coagulopathy, but appears to be insufficient in the previous reports.^[3,8]^ The present patient started her treatment with continuous heparin infusion followed by DOACs because she declined continuous heparin therapy in the outpatient setting. Pembrolizumab supported by anti-coagulation therapy was efficacious with no recurrence of thrombosis.

This is the first report of acute thrombosis as an irAE associated PD-1 pathway inhibitors including pembrolizumab in lung cancer. Inflammation from reinvigoration of T cells by pembrolizumab could bring on thrombosis. For mitigating severity of acute thrombosis, its early detection and treatment is critical.

## Author contributions

**Conceptualization:** Kei Kunimasa.

**Data curation:** Kei Kunimasa, Kazumi Nishino, Madoka Kimura, Takako Inoue, Motohiro Tamiya.

**Formal analysis:** Kazumi Nishino.

**Supervision:** Toru Kumagai, Fumio Imamura.

**Writing – original draft:** Kei Kunimasa, Fumio Imamura.

**Writing – review & editing:** Fumio Imamura.
